# Intermuscular Coherence Between Surface EMG Signals Is Higher for Monopolar Compared to Bipolar Electrode Configurations

**DOI:** 10.3389/fphys.2018.00566

**Published:** 2018-05-17

**Authors:** Maurice Mohr, Tanja Schön, Vinzenz von Tscharner, Benno M. Nigg

**Affiliations:** ^1^Human Performance Laboratory, Faculty of Kinesiology, University of Calgary, Calgary, AB, Canada; ^2^University of Applied Sciences Technikum Wien, Vienna, Austria

**Keywords:** muscle synchronization, surface electromyography, motor unit synchronization, motor unit control, motor control, quadriceps muscle, squatting exercise

## Abstract

**Introduction:** The vasti muscles have to work in concert to control knee joint motion during movements like walking, running, or squatting. Coherence analysis between surface electromyography (EMG) signals is a common technique to study muscle synchronization during such movements and gain insight into strategies of the central nervous system to optimize neuromuscular performance. However, different assessment methods related to EMG data acquisition, e.g., different electrode configurations or amplifier technologies, have produced inconsistent observations. Therefore, the aim of this study was to elucidate the effect of different EMG acquisition techniques (monopolar vs. bipolar electrode configuration, potential vs. current amplifier) on the magnitude, reliability, and sensitivity of intermuscular coherence between two vasti muscles during stable and unstable squatting exercises.

**Methods:** Surface EMG signals from vastus lateralis (VL) and medialis (VM) were obtained from eighteen adults while performing series of stable und unstable bipedal squats. The EMG signals were acquired using three different recording techniques: (1) Bipolar with a potential amplifier, (2) monopolar with a potential amplifier, and (3) monopolar electrodes with a current amplifier. VL-VM coherence between the respective raw EMG signals was determined during two trials of stable squatting and one trial of unstable squatting to compare the coherence magnitude, reliability, and sensitivity between EMG recording techniques.

**Results:** VL-VM coherence was about twice as high for monopolar recordings compared to bipolar recordings for all squatting exercises while coherence was similar between monopolar potential and current recordings. Reliability measures were comparable between recording systems while the sensitivity to an increase in intermuscular coherence during unstable vs. stable squatting was lowest for the monopolar potential system.

**Discussion and Conclusion:** The choice of electrode configuration can have a significant effect on the magnitude of EMG-EMG coherence, which may explain previous inconsistencies in the literature. A simple simulation of cross-talk could not explain the large differences in intermuscular coherence. It is speculated that inevitable errors in the alignment of the bipolar electrodes with the muscle fiber direction leads to a reduction of information content in the differential EMG signals and subsequently to a lower resolution for the detection of intermuscular coherence.

## Introduction

The vasti muscles have to work in concert to control knee joint motion and maintain balance of the body during movements such as walking, running, and squatting. Coherence analysis between surface EMG signals from synergistic muscles is a common technique to study intermuscular synchronization and gain insight into strategies of the central nervous system to control the execution of such motor tasks (Farmer et al., 1993; Semmler, 2002). Specifically, previous researchers have used EMG-EMG coherence analyses to elucidate the functional role of intermuscular synchronization, e.g., by investigating its task-dependent property for different motor tasks (Gibbs et al., 1995; Huesler et al., 1998; Kilner et al., 1999; Clark et al., 2013; van Asseldonk et al., 2014; von Tscharner et al., 2014; Mohr et al., 2015; Reyes et al., 2017) or changes in coherence during fatiguing exercises (Boonstra et al., 2008; Kattla and Lowery, 2010; Chang et al., 2012; McManus et al., 2016). These studies suggest that the neuromuscular system adjusts the degree of intermuscular synchronization based on the physical and possibly psychological demands of the movement task. However, some disagreement exists regarding the direction of change in intermuscular synchronization between different movement tasks. For example, higher and lower coherence has been reported to be necessary for balancing movements, which require individual muscle control compared to movements that are stable and require synergistic muscle control (Gibbs et al., 1995; Mohr et al., 2015; Reyes et al., 2017).

When comparing the observed EMG-EMG coherence between multiple studies, it is obvious that the magnitude of coherence as well as the frequency bands where coherence is present can be vastly different. Conceptually, there are three reasons for why previous studies show a large variation in coherence outcomes: First, different EMG recording systems were used (e.g., monopolar vs. bipolar EMG), second, different EMG signal processing techniques were applied, and/or third, the investigated motor tasks and involved muscles were governed by different neuromuscular control strategies leading to different levels of intermuscular synchronization. While many discrepancies between studies can likely be explained by the second and/or third aspect, some studies show considerable differences in coherence despite using the same analysis approaches and despite investigating the same muscles during similar tasks. Therefore, this study will address the first aspect – the influence of the EMG recording system on intermuscular coherence.

For example, Chang et al. (2012) showed that the intermuscular coherence between the vastus medialis and lateralis during a single-leg step-up task is generally lower than 0.5 across frequencies and muscle pairs. In contrast, Mohr et al. (2015) reported EMG-EMG coherence between the vasti muscles during a single-leg squat of generally higher than 0.5 and for a wider range of frequencies up to 80–100 Hz. The major difference between these studies is the use of bipolar and monopolar EMG recording systems, respectively. The rationale for the use of a monopolar over a bipolar EMG amplifier is twofold: First, monopolar EMG avoids the inherent limitation of bipolar EMG systems that the bipolar electrodes must be aligned with the muscle fiber direction. Second, due to differential amplification, bipolar EMG leads to a higher spatial selectivity while monopolar surface EMG provides a more ‘global’ view on the activity of a muscle (De Luca and Merletti, 1988). Although high spatial selectivity of bipolar EMG may be beneficial when trying to investigate the behavior of individual motor units (Reucher et al., 1987), global information on the activity of two muscles may be desired when investigating intermuscular synchronization at a whole muscle level.

The underlying concept of the bipolar technique is to detect the same motor unit action potentials twice but spatially shifted along the muscle. Then, differential amplification of these two signals leads to a reduction of noise that is common to both electrodes while the signal of interest is retained, i.e., the differential of the summed motor unit action potentials (Basmajian, 1985). This concept relies on the assumption that bipolar electrodes can in fact be aligned with the muscle fiber direction. However, most muscles fibers are oriented at a three-dimensional pennation angle with respect to the aponeurosis and the skin surface, which may change as a function of joint position and muscle force (Wickiewicz et al., 1983; Friederich and Brand, 1990; Rutherford and Jones, 1992; Merletti et al., 2001; Rainoldi et al., 2001). Even if the investigator can achieve a good alignment of the electrodes before the measurement, the assumption that the bipolar electrodes remain aligned with the muscle fiber direction during movements that involve muscle length and force changes does not hold. Bipolar electrode alignment error can alter the amplitude and frequency content of the differential EMG signal in an unknown and unpredictable way (von Tscharner, 2014), which may reduce the ability of this technology to detect intra- and intermuscular coherence.

In contrast, monopolar EMG measurements do not require electrode alignment, represent the entire information about motor unit activity near the measurement point and may thus be more suitable to study intermuscular synchronization. Accordingly, monopolar EMG has been successfully applied to resolve the task-dependent property of intermuscular synchronization between isometric and dynamic squats with a high sensitivity (Cohen’s *d* = 2.3, re-computed from Mohr et al., 2015). To the best knowledge of the authors, the effect of monopolar vs. bipolar EMG measurements on the analysis of EMG-EMG coherence is currently unknown. Based on the above argument, however, it is speculated that the disruption of information in bipolar EMG recordings may lead to a lower resolution to detect high EMG-EMG coherence between muscles and explain the discrepancies between previous studies. Despite these possible advantages of monopolar surface EMG, the technique is not commonly used in biomechanical and neuromuscular investigations. This is due to the susceptibility of monopolar surface EMG to noise from stray-potentials, movement artifacts, and possibly cross-talk due to low spatial selectivity (De Luca and Merletti, 1988; von Tscharner et al., 2013), which may compromise the reliability of a monopolar system.

In addition to using a monopolar electrode configuration, Mohr and colleagues obtained EMG signals via a recently developed current amplifier in contrast to the classic EMG potential amplifier (von Tscharner et al., 2013). The main difference is that the current amplifier injects or withdraws charges at the skin surface above the active muscle to keep all measurement points at ground potential while the potential amplifier relies on a potential at the skin surface with respect to the ground electrodes. The concept of the current amplifier has the advantage that inter-electrode currents are avoided, which enables EMG measurements during conditions when the inter-electrode impedance is largely reduced, e.g., when sweat builds up on the skin or even during extreme conditions such as during underwater measurements (Whitting and von Tscharner, 2014). Furthermore, current measurements may be more sensitive to the EMG signals at higher frequencies compared to measurements from potential amplifiers and may therefore be more suitable to explore coordinated motor unit activity at frequencies beyond the typically investigated beta and gamma bands (>60 Hz) (von Tscharner et al., 2013).

In summary, there are well-founded arguments for the use of monopolar amplifiers and current measurements instead of the traditional bipolar potential measurements when investigating EMG-EMG coherence. However, the effect of monopolar vs. bipolar electrode configurations and potential vs. current EMG recording techniques on the magnitude and frequency of intermuscular coherence has not been systematically investigated. Similarly, it is unknown whether one of these EMG recording techniques can more reliably detect intermuscular coherence or if one is more sensitive to detecting a change in intermuscular coherence between different movement tasks.

Therefore, the first objective of this study was to compare intermuscular coherence of vastus lateralis and medialis surface EMG signals during a dynamic, bipedal squatting task between three different EMG recording techniques: Bipolar potentials, monopolar potentials, and monopolar currents.

The second objective was to compare these three techniques regarding their reliability when repeatedly assessing a stable squatting task and their sensitivity to detecting a change in intermuscular coherence between squatting on a stable vs. unstable surface.

It was hypothesized that:

(1) VL-VM intermuscular coherence would be higher for monopolar EMG signals compared to bipolar signals, and
(2) All three recording techniques would be sensing a lower VL-VM intermuscular coherence during unstable compared to stable squatting although monopolar systems would show a reduced reliability between similar squatting trials.

## Materials and Methods

### Participants

Eighteen healthy, male (*n* = 14) and female (*n* = 4) participants (mean ± SD; age 26 ± 5 y; height 175 ± 6 cm; mass 69 ± 7 kg) volunteered to participate in this study. This study was carried out in accordance with the guidelines of the University of Calgary’s Conjoint Health Research Ethics Board. The protocol was approved by the University of Calgary’s Conjoint Health Research Ethics Board (#REB17-0210). All subjects gave written informed consent in accordance with the Declaration of Helsinki.

### Study Design

Each participant completed a total of six squatting trials, three trials were recorded with the monopolar current amplifier system and three trials were recorded with the monopolar potential amplifier system (**Table 1**). The bipolar potential signals were computed from the monopolar signals following data acquisition (see section “EMG potential measurements”). The order of recording systems was balanced randomized. The order of trials was kept constant and consisted of two trials of squatting on a stable surface and one trial of squatting on an unstable surface. The protocol included two trials of stable squatting to determine the between-trial reliability of intermuscular coherence and one trial of unstable squatting to determine the sensitivity of the three systems.

**Table 1:** Design of experimental procedures.

Amplifier	Configuration	Trial 1	Trial 2	Trial 3
Potential	Monopolar	Stable	Stable	Unstable
	Bipolar			
Current	Monopolar	Stable	Stable	Unstable


### Squatting Tasks

**FIGURE 1:**
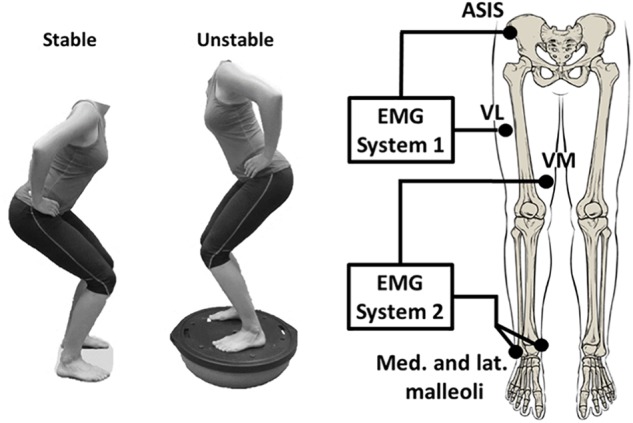
Stable vs. unstable squat (left); conceptual EMG recording set-up (right).

During each trial, participants performed a series of squats down to a knee flexion angle of 70 degrees (0 degrees represents full extension) for a duration of 90 s. The distance between the participants’ feet was self-selected and kept constant throughout all trials but had to be at least shoulder wide apart (**Figure 1**). Stable squatting trials were performed on the laboratory floor while unstable squatting trials were completed on the flat side of a BOSU ball (**Figure 1**). For all trials, the squatting speed was set to 20 squats per minute and controlled for by using a metronome at 40 bpm yielding a total of 30 squats per trial that were used for data analysis. In order to ensure consistent knee flexion angles at the lowest squat position, participants were given visual real-time feedback from a one-dimensional electrogoniometer (Biometrics Ltd., United Kingdom) taped across the anterior side of their knee joint. Each participant was given one initial practice trial to familiarize with the equipment and squatting speed. For each following trial, the EMG recording system was started once the participant had found the correct squatting rhythm.

### EMG Electrode Placement

In order to obtain surface EMG signals from VM and VL, the skin surface above the muscles was shaved, slightly abraded with sand paper and cleaned with alcohol wipes to ensure high signal conductivity. Two Ag-AgCl electrodes (10 mm diameter, 20 mm inter-electrode distance, Norotrode Myotronics-Noromed Inc., United States) in a bipolar configuration were placed over the muscle bellies of VM and VL using the following procedure. First, the electrode positions and orientations on VM and VL were located and marked according to EMG sensor locations described in SENIAM guidelines (Hermens et al., 1999). Next, an ultrasound machine was used to verify that the marked electrode locations were within the proximal-distal and medio-lateral boundaries of the muscles while the participants where performing a static squat at 45 degrees of knee flexion.

### EMG Recording Systems

Electromyography recordings of each muscle were obtained using two separate recording systems with separate ground electrodes, data acquisition cards (12-bit A/D converter, National Instruments, Austin, TX, United States), and battery powered laptops. Thus, the systems consisted of two electronically separated circuits to avoid hardware-based crosstalk (Mohr et al., 2015). In system 1, EMG signals of VL were recorded with reference to two ground electrodes placed side by side on the right anterior superior iliac spine. In system 2, EMG signals of VM were recorded with reference to two ground electrodes placed on the medial and lateral malleoli (**Figure 1**). Two ground electrodes were used in each system to improve the stability of the ground potential and to further reduce the resistivity to the returning currents. Each electrode was connected to an extension lead and then fixed in place using adhesive stretch tape. This step was necessary to ensure that the electrode-skin connection was kept constant throughout the protocol when switching between the current and potential measurements. The two recording systems of VL and VM were synchronized using a custom-built device that simultaneously transmitted a pulse to both systems upon pressing a button at the beginning and end of each measurement.

### EMG Potential Measurements

Two monopolar EMG potentials were recorded from each muscle using a total of four differential amplifiers at a sampling frequency of 2400 Hz with a hardware-based bandpass filter between 10–500 Hz (Biovision, Wehrheim, Germany). The positive input of the amplifiers was connected to one of the two electrodes placed on each muscle and the negative input was connected to the respective ground. In this configuration, one can use a differential amplifier to record monopolar EMG potentials. Bipolar EMG potentials for VM and VL were computed following data acquisition by calculating the difference between the two monopolar EMG potentials obtained from each muscle (proximal – distal electrode). This approach was selected to compare intermuscular coherence between monopolar and bipolar EMG potential measurements that were obtained from the same squatting trial. A pilot experiment was conducted where bipolar EMG potentials directly recorded from the VL with a single differential amplifier were compared with the computed bipolar EMG potentials as explained above. The power spectra of a 60 s isometric squat were virtually identical between the two methods, thus verifying the validity of the approach.

### EMG Current Measurements

Monopolar EMG currents were recorded from the proximal electrode on each muscle using a previously described and validated current amplifier at a sampling frequency of 2400 Hz and a hardware-based bandpass filter between 10–500 Hz (von Tscharner et al., 2013; Mohr et al., 2015).

### EMG Signal Analysis

#### Filtering

Goniometer data were low-pass filtered (cut-off frequency of 1 Hz) using a wavelet-based filter method. The 60 Hz line-frequency contamination was removed from all monopolar EMG signals by applying a line-frequency averaging method and a line filter. In short, this procedure allows to subtract the average line-frequency contamination from the EMG signal without inducing a notch in the EMG power spectrum at 60 Hz (see von Tscharner et al., 2013 for further details). Removing the line-frequency from the signals avoided an artificial intermuscular coherence at 60 Hz. The lowest frequency that was considered for this analysis was 10 Hz, which is given by the 10–500 Hz bandpass filter of the EMG amplifiers and by the notion that the power density function of the surface EMG signal has negligible contributions below 10 Hz (Merletti, 1999).

#### Sequencing

**FIGURE 2:**
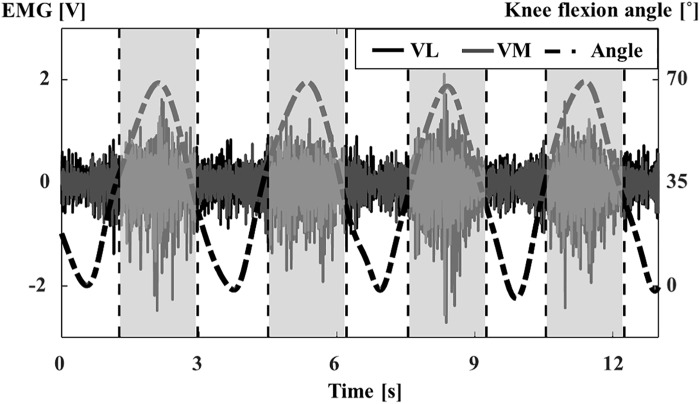
Procedure to separate individual data sequences according to peaks in the knee flexion angle. VL (black, solid line) and VM (gray, solid line) filtered EMG signals recorded with the monopolar potential system and corresponding knee flexion angle (black, dash-dot line). Dashed vertical lines to the left and right of peaks in the knee flexion angle trace indicate the boundaries of individual data sequences (shaded) that were used for further analysis.

For each squatting trial, the signals were separated into 30 sequences of 4096 samples (1.7 s) according to peaks in the goniometer signal that represented the time points of highest knee flexion, i.e., the deepest positions during the squats. While these sequences contained the majority of the EMG power during the squats (**Figure 2**), the exact sequence size facilitated using a fast Fourier transform (FFT) during the analysis.

#### Power and Coherence

The FFT of the unrectified EMG signals was computed for each data sequence, leading to a frequency resolution of 0.6 Hz. The power spectra for each muscle and trial were determined by multiplying the FFT of each sequence with its complex conjugate and averaging across all data sequences. Intermuscular coherence as a function of frequency λ (coherence spectrum) between VL and VM EMG signals for one given squatting trial was computed from the average cross-spectra normalized by the corresponding power spectra across *s* = 30 data sequences (Rosenberg et al., 1989):

$$\text{coherence}(\lambda )=\frac{{|\overset{¯}{F_{{VL}_{s}}(\lambda )\cdot F_{{VM}_{s}}\left(\lambda )*\right|}}^{2}}{(\overset{¯}{F_{{VL}_{s}}(\lambda )\cdot F_{{VL}_{s}}\left(\lambda \right)}*)\cdot \overset{¯}{\left(F_{{VM}_{s}}(\lambda )\cdot F_{{VM}_{s}}(\lambda )*\right)}}$$

For each trial and participant, the average coherence was computed as the mean of the coherence spectrum between 10–60 Hz. The frequency range of 10–60 Hz was chosen since the coherence in this range was highest across all trials and participants and since it spans frequencies in the beta (15–30 Hz) and gamma (30–60 Hz) bands, at which intermuscular coherence is typically reported in the literature (Clark et al., 2013; Marchis et al., 2015; Pizzamiglio et al., 2017).

To assess the possible influence of cross-talk between the vasti muscles on the level of intermuscular coherence measured with different recording systems, a simple simulation was performed. From previous studies it was estimated that in the monopolar electrode configuration, there may be an additional 10% of cross-talk compared to the bipolar configuration due to the absence of spatial filtering (Farina et al., 2002). Therefore, a pair of simulated monopolar VL and VM signals was computed from the respective bipolar EMG signals by adding the VL signal multiplied by a factor of 0.1 to the VM signal and vice versa. The coherence analysis as described above was then repeated for these computed signals with simulated cross-talk.

#### EMG Intensity

In order to determine whether the level of VL and VM muscle excitation changed between the stable and unstable squatting condition, the overall EMG intensity was determined using a wavelet transform. In short, a filter bank of 30 non-linearly scaled wavelets specifically designed for EMG analysis was used to decompose the raw EMG signals into the time-dependent power in each of the frequency bands (wavelets) (von Tscharner, 2000). For this analysis, powers from twenty wavelets with center frequencies between 10–300 Hz were summed to derive the total power. The square root of the total power yields the total EMG intensity, a close approximation of the frequently used EMG root mean square (von Tscharner, 2000). The overall EMG intensity of VL and VM representing the average level of muscle excitation was calculated as the sum of the total EMG intensity for each individual squat. For each recording system separately, the overall EMG intensities were normalized to the maximum overall EMG intensity obtained across all 90 squats (3 trials of 30 squats). Finally, the normalized EMG intensities were averaged across the 30 squats for each trial to derive one normalized, mean overall EMG intensity for each trial, system, muscle and participant.

### Statistical Analysis

For each trial (stable 1, stable 2, unstable) and recording technique (bipolar potential, monopolar potential, monopolar current), the mean and standard deviation of the power and coherence spectra, average coherence values, and normalized overall EMG intensities were computed across 16 participants. In addition, the mean and standard deviation of the average coherence values for the simulated monopolar signals were determined to investigate a possible influence of cross-talk. Two male participants had to be excluded from the analysis as they were not able to perform the unstable squatting trials without help from the investigator. A two-way repeated measures ANOVA with the within-subject factors ‘trial’ and ‘recording technique’ was performed to detect significant main and interaction effects on the average coherence. Mauchly’s test of sphericity was used to test the assumption of sphericity. If the assumption of sphericity was violated, the Huynh–Feldt correction was used and reported. Bonferroni-corrected *post hoc* tests were carried out to determine pairwise comparisons of coherence between individual trials and recording techniques. To investigate a possible effect of squatting technique on the level of muscle excitation, separate two-way repeated measures ANOVAs with the within-subject factors ‘trial’ and ‘recording technique’ were performed for the overall EMG intensities of the two muscles VL and VM. All statistical tests were carried out at a significance level of 0.05 using IBM SPSS statistics (v. 24; SPSS Inc., Chicago, IL, United States).

The reliability of the average VL-VM coherence was determined for each recording technique using the first and second stable squatting trial. Relative reliability was computed using the intra-class correlation coefficient [ICC, model 3 (two-way mixed effects, absolute agreement), type 1] and the corresponding 95% confidence intervals (Shrout and Fleiss, 1979; McGraw and Wong, 1996; Koo and Li, 2016). Absolute reliability was determined using the standard error of measurement (SEM) according to equation (2):

$$SEM=\sqrt{{MS}_{E}}$$

where *MS*_E_ is the error term obtained from the ANOVA table of the ICC calculations (Eliasziw et al., 1994). The SEM represents the random error of the obtained scores in comparison to the ‘true’ scores in the original units of measurement with the assumption that there is no systematic bias between the measurements. To decide whether an observed change in the obtained scores can be considered ‘true’ change, the SEM can be used to derive the minimal detectable change (MDC), according to equation (3):

$$MDC=1.96\sqrt{2}\,\;SEM$$

The sensitivity of the three recording systems to a change in the average VL-VM coherence when changing from the stable to the unstable squatting condition was assessed according to Cohen’s *d* as a measure of effect size. Specifically, the effect size for each system was determined as the mean of the differences between the two conditions (unstable – stable) divided by the standard deviation of the differences. Values for Cohen’s *d* of greater than 0.8 represent large effects (Cohen, 1992).

## Results

### Mean Power and Coherence Spectra

**FIGURE 3:**
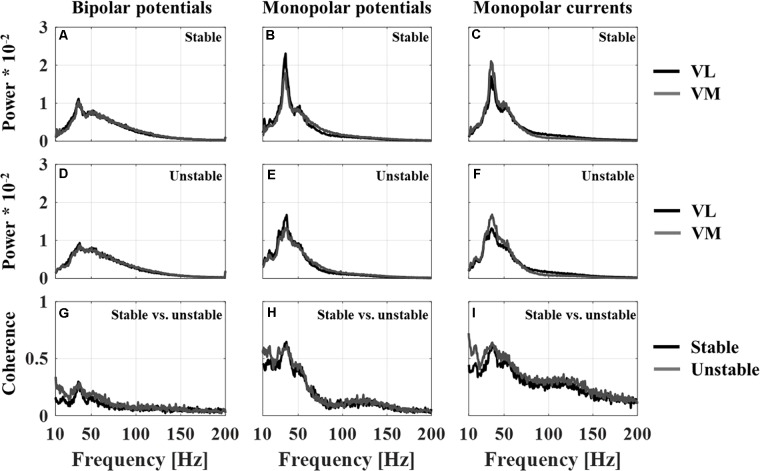
Average of the normalized power spectra across all subjects (*n* = 16) for vastus lateralis and medialis for each recording technique (BP – bipolar potentials, MP – monopolar potentials, MC – monopolar currents) for the stable squat **(A–C)** and unstable squat **(D–F)**; Average of the coherence spectra across all subjects (*n* = 16) between vastus lateralis and medialis for each recording technique and squatting condition **(G–I)**.

**Figure 3** displays the average power spectra and coherence spectra for all recording techniques and the stable and unstable squatting condition. The average VL-VM coherence spectra are clearly reduced when obtained from bipolar compared to monopolar recordings (**Figures 3G–I**). Although the coherence spectra of monopolar potentials and currents generally show a similar shape, the spectra obtained from monopolar currents demonstrate a higher coherence for frequencies above 80 Hz. In addition, it can be observed that the coherence during unstable squatting is larger compared to stable squatting, particularly for frequencies below 40 Hz.

While the power spectra for VL and VM show a similar pattern, the spectra show a different shape when comparing the bipolar and monopolar recording techniques. Specifically, for monopolar recordings the power spectra demonstrate a pronounced peak in the frequency range of 30–50 Hz, which is much less visible in spectra obtained from bipolar recordings. It is also within this frequency band, that a high coherence was observed in the coherence spectra. Similarly, the magnitude of this 30–50 Hz peak is reduced during the unstable compared to the stable squatting condition (**Figures 3A–F**).

### Average Coherence

There were significant main effects of ‘trial’ [*F*(1.44,21.59) = 18.24, *p* < 0.001] and ‘recording technique’ [*F*(3,45) = 61.3, *p* < 0.001] on the average coherence. There was no significant interaction term between ‘trial’ and ‘recording technique’ [*F*(3.86,57.9) = 1.79, *p* = 0.144].

**FIGURE 4:**
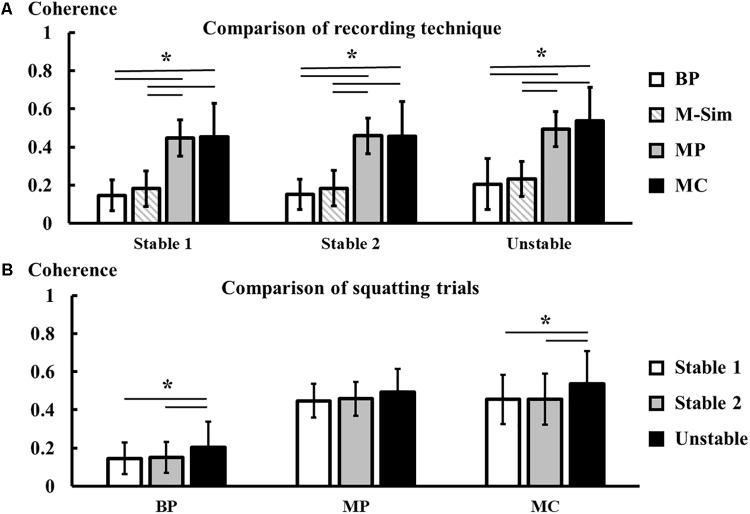
Comparison of average coherence (mean ± SD, *n* = 16) between recording techniques (BP – bipolar potentials, M-Sim – simulated monopolar potentials, MP – monopolar potentials, MC – monopolar currents) **(A)**, and comparison of average coherence between squatting trials **(B)**. Asterisks mark statistically significant differences between conditions at α = 0.05.

Regarding the first study objective, the *post hoc* comparisons indicated that VL-VM intermuscular coherence was significantly reduced by more than 50% for bipolar potential measurements compared to monopolar potential and monopolar current measurements for each individual squatting trial. There were no significant differences in average coherence between the monopolar potential and current measurements. Further, there were no significant differences in average coherence between the bipolar measurements and the simulated monopolar signals with added cross-talk (**Figure 4A**).

Coherence was significantly higher during the unstable squatting trial compared to both stable squatting trials for the bipolar potential and monopolar current measurements. Despite a similar trend, there were no statistically significant differences in the average coherence between squatting trials for the monopolar potential measurements (**Figure 4B**).

Regarding the second study objective, the reliability and sensitivity of the three recording systems are displayed in **Table 2**. For all recording systems, there were no average differences between the first and second stable squatting trial (**Figure 4B**). For both potential measurements, the lower bounds of the ICC 95% confidence intervals were above 0.75, indicating good relative reliability (Koo and Li, 2016). The monopolar current measurement showed excellent relative reliability [ICC = 0.98 (0.94,0.99)]. The MDC in average VL-VM coherence during squatting was between 0.06 for the bipolar potentials and 0.07 for the two monopolar systems. Only for the monopolar current measurements, the mean difference between the unstable and stable squatting condition (trial 3 – trial 1) exceeded the MDC. Similarly, the monopolar current measurements showed the highest effect size (Cohen’s *d* = 1.34), followed by the bipolar (0.91) and monopolar potentials (0.63).

**Table 2:** Reliability and sensitivity of average coherence outcomes.

	Reliability				Sensitivity		
		
System	Relative		Absolute		Unstable – Stable		Cohen’s d
				
	ICC	95% CI	SEM	MDC	Mean	SD	
Bipolar potential	0.93	(0.81,0.97)	0.02	0.06	0.06	0.07	0.91
Monopolar potential	0.92	(0.80,0.97)	0.03	0.07	0.05	0.07	0.63
Monopolar current	0.98	(0.94,0.99)	0.03	0.07	0.08	0.06	1.34


### EMG Overall Intensity

**FIGURE 5:**
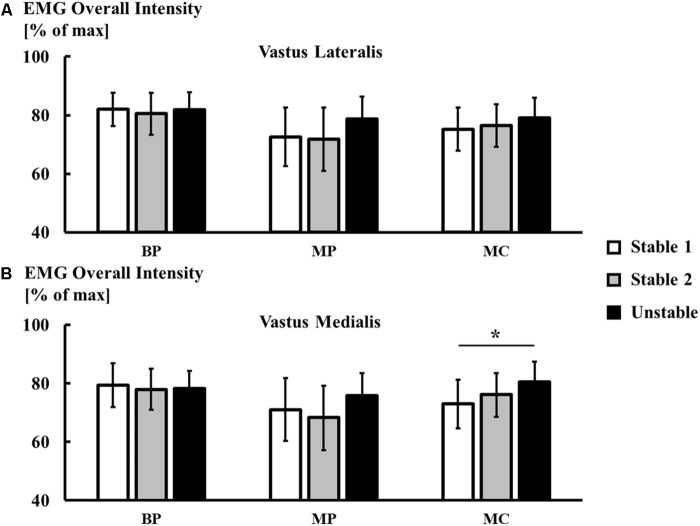
Comparison of normalized overall EMG intensity (mean ± SD, *n* = 16) between recording techniques (BP – bipolar potentials, MP – monopolar potentials, MC – monopolar currents) for the vastus lateralis **(A)**, and vastus medialis **(B)**. Asterisks mark statistically significant differences between conditions at α = 0.05.

**Figure 5** shows the average, normalized overall EMG intensity of VL and VM during all three squatting trials. For both muscles, there was a significant interaction effect between ‘trial’ and ‘recording technique’ on the overall EMG intensity [VL: *F*(2.78, 41.75) = 3.07, *p* = 0.041; VM: *F*(3.27,49.02) = 4.39, *p* = 0.007]. *Post hoc* comparisons showed that on average, there were no significant differences in EMG intensity between the stable and unstable squatting condition for the bipolar system. For all monopolar recordings, there was a small trend for an average percentage increase of about 10% during the unstable compared to stable squatting condition. The average increase in overall EMG intensity in the unstable vs. stable condition only reached statistical significance for the current measurements of the vastus medialis (trial 1 vs. 3, *p* = 0.028). The large standard deviations in **Figure 5** indicate that there was a high degree of variability between the individuals regarding which of the three squatting trials showed the highest overall EMG intensity.

## Discussion

This study aimed to investigate the effect of different EMG recording techniques on the magnitude, reliability, and sensitivity of intermuscular coherence during dynamic squatting tasks. Our first hypothesis that intermuscular coherence would be higher when computed from monopolar compared to bipolar EMG signals was confirmed by the result that the average coherence for bipolar potential measurements was significantly reduced compared to the monopolar potential and current measurements. The second hypothesis that all three systems would be sensitive to a lower VL-VM coherence during unstable vs. stable squatting was not supported by the findings that (1) the monopolar potential recordings showed low sensitivity to the change in coherence between the two squatting conditions and (2) the average coherence was in fact higher during unstable compared to stable squatting.

### Recording Techniques – Bipolar vs. Monopolar

The reduction in coherence for bipolar compared to monopolar EMG recordings may have two possible reasons: (1) the reduction or disruption of amplitude and frequency information within the bipolar signals that arose from electrode alignment and position errors and subsequent differential amplification, and (2) less cross-talk in bipolar recordings due to spatial filtering and higher spatial selectivity.

When a muscle is activated, motor unit action potentials travel along the muscle fibers, starting from the innervation zone and ending at the muscle insertion or origin (Basmajian, 1985). For bipolar EMG measurements, two adjacent electrodes are applied between innervation zone and tendon and in alignment with the muscle fiber direction to detect the same motor unit action potentials twice but spatially shifted along the muscle. Subtracting the signals from these two monopolar EMG signals yields a single-differential bipolar EMG signal, which has the advantage that common noise under both electrodes is reduced (Gallina et al., 2013). Consequently, bipolar EMG measurements have been state-of-the-art in investigating muscle activation patterns during movement and have frequently been used to study intermuscular synchronization (Gibbs et al., 1995, 1997; Kilner et al., 1999; Halliday et al., 2003; Boonstra et al., 2008; Kattla and Lowery, 2010; Reyes et al., 2017). During movements such as squatting, however, the fiber direction and location of the innervation zone of the quadriceps muscles with respect to the electrode position on the skin are functions of the knee angle and quadriceps muscle force (Rutherford and Jones, 1992; Gallina et al., 2013). Similarly, the innervation zone has been shown to move with respect to the skin as a function of knee angle (Rainoldi et al., 2000; Gallina et al., 2013). In consequence, bipolar electrodes cannot be properly aligned with the muscle fiber direction and the bipolar EMG signal will likely represent a combination of (1) the differential between propagating motor unit action potentials from the same motor units recorded twice at different locations along the muscle fiber direction and (2) the differential between motor unit action potentials that originate from different motor units (von Tscharner et al., 2013). The ratio of these differentials depends on the geometry of the muscle as well as electrode placement and will change with the pennation angle throughout a movement. It is well known that a portion of motor units within one muscle are synchronized in time, i.e., intramuscular synchronization, with an accuracy of more than 5 ms (Sears and Stagg, 1976; Bremner et al., 1991; De Luca et al., 1993). Therefore, it is likely that signals recorded by the electrodes in a bipolar measurement setup are highly correlated and are either eliminated or at least disrupted in an unspecific and unpredictable way by the common mode rejection of the amplifier (von Tscharner, 2014). In consequence, only the signals that are uncorrelated between adjacent electrodes, i.e., not synchronized, are retained in bipolar measurements.

Evidence for this assumption can be seen in the average power spectra that were obtained using the monopolar and bipolar recording systems in this study. The monopolar recordings demonstrate a pronounced peak in the power spectrum between frequencies of 30–50 Hz. This 40 Hz peak in the EMG power spectrum of dynamic tasks has been observed previously and has been connected to rhythmic bursts of clustered motor unit activity, where multiple motor units are firing within a short time window of 10 ms (Yao et al., 2000; Maurer et al., 2013; Asmussen et al., 2018). If the two electrodes of a bipolar amplifier are recording motor unit action potentials from different motor units that are virtually firing at the same time, the common mode rejection would likely remove a significant amount of this information and explain why the 40 Hz peak is absent or much reduced in amplitude in the power spectra obtained from bipolar recordings.

Both intra- and intermuscular synchronization of motor units as measured by EMG-EMG coherence have been speculated to originate primarily from common, or shared inputs of the corticospinal tract to the respective motoneuron pools (Bremner et al., 1991; Farmer et al., 1993; Lowery et al., 2007). In consequence, the degree of intra- and intermuscular synchronization is most likely correlated. If the bipolar EMG signal from one muscle only contains information about uncorrelated motor unit activity, as described above, it will be more difficult to detect intermuscular synchronization between different muscles. In contrast, monopolar EMG recordings contain the entire signal information and inherently do not need to be aligned with the muscle fiber direction. Therefore, it is speculated that the reduced or disrupted information within the bipolar EMG signal is the theoretical basis for the reduced VL-VM coherence in comparison to monopolar signals seen in this study.

The second possible origin of the difference in intermuscular coherence observed between the recording systems is a varying influence of cross-talk. Cross-talk ratios of about 10% were reported between the VL and VM during isometric knee extensions (Farina et al., 2002). Specifically, it has been shown that cross-talk was reduced in surface EMG signals from the thigh muscles when they were obtained using a double-differential vs. a single-differential recording technique (De Luca and Merletti, 1988; Farina et al., 2002). The reduction of cross-talk is most likely due to an increase in spatial selectivity of the EMG system when using double-differential amplification (Reucher et al., 1987). Albeit not systematically investigated to date, it can be speculated that monopolar surface EMG signals may thus contain more cross-talk components compared to bipolar single-differential signals. Cross-talk between EMG recordings of adjacent muscles can artificially inflate EMG-EMG coherence and should therefore be carefully addressed as a potential confounding factor in this study (Grosse et al., 2002; Halliday et al., 2003). There are three reasons why the influence of cross-talk on the findings of this study is likely small. First, the EMG measurement set-up of this study was carefully designed to record VL and VM signals using two electronically separated circuits with separate grounds to exclude the possibility of hardware-based cross-talk (Mohr et al., 2015). Second, when measuring intermuscular EMG-EMG coherence, significant cross-talk between the two muscles of interest typically leads to a resulting coherence spectrum that shows high values across a broad range of frequencies, spanning almost the entire EMG bandwidth (Grosse et al., 2002; Halliday et al., 2003). This was not observed for any of the individuals tested in this study. Third and most importantly, we used a simple simulation to investigate the possible influence of an additional 10% of cross-talk components in monopolar compared to bipolar EMG signals. Although on average, the simulated signals show a slightly higher coherence compared to the bipolar signals (see **Figure 4A**, BP vs. M-Sim), this difference was not statistically significant and cannot explain the large increase in coherence from the bipolar to the monopolar recording systems. In summary, while the presence of cross-talk can not be completely excluded in this study, cross-talk was not a major confounding factor in the comparison of monopolar vs. bipolar EMG systems.

### Recording Techniques – EMG Intensity

A second difference between the recording systems was observed for the level of muscle excitation according to the overall EMG intensity during the stable and unstable squatting exercise. While the bipolar EMG measurements of the vasti muscles did not show an average change in EMG intensity between the movement conditions, the monopolar recording systems showed a small, average increase in EMG intensity for both VL and VM during the unstable squat. This is in accordance with a previous study showing no or only a small percentage increase (<10%) in thigh muscle activity when switching from squatting on a stable to squatting on an unstable surface (Anderson and Behm, 2005). The discrepancy between the monopolar and bipolar recording systems could originate from additional synchronized inputs that the vasti muscles received from the central nervous system during the unstable squat as suggested by the corresponding increase in VL-VM coherence during this exercise. Such synchronized motor unit activity would increase the overall EMG intensity (Yao et al., 2000; Asmussen et al., 2018) but may not be detected by the bipolar EMG recording system due to the elimination or reduction of common input signals as explained above.

### Recording Techniques – Potentials vs. Currents

A third difference between the recording systems was observed between the coherence spectra obtained using the monopolar current compared to the potential amplifiers. Specifically, the current amplifier detected a higher magnitude of coherence for frequencies above 80 Hz compared to the potential amplifiers. The presence of high-frequency intermuscular coherence has been reported between EMG signals for upper and lower limb muscles (Chang et al., 2012; Marchis et al., 2015; Mohr et al., 2015; Pizzamiglio et al., 2017). Intermuscular coherence within the gamma band (30–60 Hz) and higher frequencies has been speculated to represent a coupled, descending motor command to muscles involved in movement tasks that require dynamic modulation of muscle force for error correction – such as squatting on an unstable surface in the current study (Pizzamiglio et al., 2017). For these force modulations, it may be preferable for the central nervous system to primarily activate fast motor units due to their ability to generate higher forces and faster conduction velocities (Milner-Brown et al., 1973; Wakeling and Syme, 2002; Hodson-Tole and Wakeling, 2009). In parallel, it has been suggested that faster motor units generate motor unit action potentials that contribute high-frequency components to the EMG signal (Wakeling and Rozitis, 2004), which could explain the second, smaller peak in the coherence spectra at frequencies above 100 Hz seen in this study (see **Figures 3H,I**). However, direct evidence for a preferential recruitment of fast, large motor units for a mixed fiber type muscle is currently not available. Nevertheless, it is unlikely that this second high-frequency coherence peak seen for the current recordings is due to a measurement artifact but it is unclear why this peak is much reduced or absent in the potential recordings.

Previously, von Tscharner et al. (2013) had observed that the monopolar current amplifier is more sensitive in detecting EMG signal power at high frequencies, which could be a reason for the higher EMG-EMG coherence at these frequencies in the current recordings. However, **Figure 3** does not show an obvious difference between the average power spectra of monopolar current and potential amplifiers at frequencies above 80 Hz. It may be that synchronized, fast motor units only have a negligible contribution to the average EMG power, which is dominated by frequencies below 80 Hz, but that they still contribute to the coherence spectrum, which is independent of signal amplitude. Further research is required to understand why the current amplifier may be more sensitive in resolving motor unit action potentials at higher frequencies.

### Stable vs. Unstable Squat

Both the bipolar potential and monopolar current system showed an average increase in VL-VM coherence during the squat on the unstable BOSU balance trainer compared to the stable squat. Albeit not statistically significant, the monopolar potential system also showed an increase toward a higher VL-VM coherence during unstable squatting. In parallel, there was no difference in intermuscular coherence between the first and second trial of stable squatting, demonstrating the absence of a possible learning effect. For both the bipolar potential and monopolar current system, the increased VL-VM coherence during unstable squatting was equal to or exceeded the respective MDC. In combination, these findings suggest that the neuromuscular strategy to control the vasti muscles changed when adding an unstable surface to the squatting exercise.

While all three recording systems indicated an average increase in VL-VM coherence between the two movement conditions, the bipolar potential and monopolar current systems were more sensitive compared to the monopolar potential system. Therefore, if researchers are interested in studying a change in intermuscular coherence between two different tasks, the bipolar potential system or monopolar current system seem to be more suitable than the monopolar potential technique.

The squatting movement on the BOSU balance trainer was selected as a task that is comparable to squatting on a stable surface in terms of joint kinematics and net force while demanding a greater involvement of the individual quadriceps muscles in maintaining postural stability. The result of a higher coherence during unstable squatting was not expected since previous investigators have reported a reduction in intermuscular coherence when performing a task that requires more individual muscle control compared to a task that requires more synergistic muscle control (Mohr et al., 2015; Reyes et al., 2017). For example, Reyes et al. (2017) demonstrated a reduction in intermuscular beta-band coherence (15–30 Hz) between a finger and a thumb muscle during a task where participants pinched an unstable spring compared to a task where a stable cylinder was compressed with a matched force. Furthermore, musicians who require more individual control of finger muscles showed a lower degree of motor unit synchronization within a finger muscle compared to weight lifters who have trained to use their finger muscles in synergy (Semmler and Nordstrom, 1998). It is questionable, however, whether vastus medialis and lateralis in this study were in fact controlled more individually by the central nervous system during the squat on the BOSU balance trainer compared to the stable squat. Anderson and Behm compared the general level of EMG intensity of vastus lateralis as well as of lower leg and core muscles between squatting on a stable vs. unstable surface (Anderson and Behm, 2005). Vasti EMG intensity was not significantly different between the two squatting conditions, which corroborates the result of this study, whereas the EMG intensity of the core and lower leg muscles was increased by up to 50% during the unstable condition. This indicates that the role of the quadriceps in maintaining postural stability during the unstable squat is small in relation to core and lower leg muscles. As a consequence, it may not be appropriate to compare the current findings with previous studies that investigate individual muscle control paradigms. Instead, the authors speculate that during both squatting exercises, the vasti muscles act as prime movers and were thus controlled as a functional unit by the central nervous system (De Luca and Erim, 2002; Anderson and Behm, 2005). During the unstable squat, the motor units of the vasti muscles may have received additional, intermittent and synchronized inputs to achieve small adjustments in the knee flexion angle trace while squatting on the BOSU ball. Furthermore, these intermittent bursts of activity may have disturbed the rhythmic, clustered motor unit activity related to the 40 Hz peak in the VL and VM power spectra and, thus explain the reduced magnitude of this peak during the unstable squat in **Figure 3**. In support of this argument, Gibbs et al. (1995) showed that the motor unit synchronization between two synergistic lower leg muscles as measured by a cross-correlation analysis was higher during a balancing standing task compared to a regular standing task and compared to voluntary contractions while lying down. It was suggested that the increase in motor unit synchronization may originate from a greater involvement of the vestibular system, specifically that the muscles received synchronized inputs from increased activity in vestibulospinal neurones. The authors speculate that a similar neuromuscular mechanism could explain the finding of higher VL-VM intermuscular coherence during the balancing task in this study.

### Reliability and Sensitivity

The question remains if one of the EMG recording techniques, bipolar vs. monopolar, is more suited to investigate EMG-EMG coherence as a measure of intermuscular synchronization. A higher coherence score alone does not necessarily indicate that the monopolar system is more suitable. Therefore, reliability and sensitivity analyses were performed to give further insight into this question. Comparing all three systems, it was observed that the coherence obtained from the monopolar currents showed the highest relative reliability between two stable squatting trials as well as the highest sensitivity when changing to unstable squatting with a large effect size of greater than one. The monopolar potential measurements, however, showed a low sensitivity and could not resolve the increase in coherence when changing between squatting conditions. This could be because monopolar potential recordings are more susceptible to stray potentials in the measurement environment and electrical noise that could contaminate the signals and reduce the system sensitivity (von Tscharner et al., 2013). The bipolar system showed good relative reliability and resolved a large effect between the stable and unstable squat, although with a lower sensitivity compared to the monopolar current system.

Therefore, when studying EMG-EMG intermuscular coherence to investigate the relative change in intermuscular synchronization between two or more movement conditions, both the bipolar potential and monopolar current systems seem to be suitable while the monopolar potential system should not be used. A monopolar current technique may be preferable over the traditional bipolar technique if (1) the muscles of interest are far enough apart that cross-talk between monopolar electrodes has a minor influence, and (2) the movement of interest does not involve impacts, e.g., walking or running. The latter would induce large motion artifacts in monopolar EMG measurements, which would produce a misleading EMG-EMG coherence.

When studying the magnitude of intermuscular coherence as a measure of the absolute degree of intermuscular synchronization between two muscles for a certain individual or a group of individuals, monopolar EMG recordings on the one hand may provide a more ‘global’ view on correlated motor unit activity at the whole muscle level. On the other hand, bipolar EMG recordings, particularly in combination with additional spatial filtering techniques or when applied as multi-electrode arrays, may provide better information on the behavior and synchronization of individual motor units. Whether one or the other technique better represents the physiological origin of correlated motor unit activity, i.e., the strength of common inputs to the motor neuron pools of two muscles, should be the focus of future studies.

## Conclusion

This study investigated the effect of three different surface EMG recording systems on the coherence between the raw EMG signals of vastus medialis and lateralis during bipedal squatting on stable and unstable surfaces. When EMG signals were obtained with the traditional bipolar potential amplifier, the magnitude of intermuscular coherence between 10–60 Hz was less than half compared to the coherence based on monopolar signals. This may be a consequence of disrupted information about motor unit activity contained in the bipolar EMG signals as a result of the elimination of common signals by the differential bipolar amplifier. A simple simulation of additional cross-talk in monopolar signals could not explain this substantial difference in coherence between the recording systems. When comparing squatting exercises on a stable and unstable surface, only the bipolar potential and monopolar current system resolved an increase in intermuscular coherence for the unstable surface, with a larger effect size for current measurements. The monopolar potential system showed low sensitivity to the change in the movement condition and should therefore not be used to determine intermuscular coherence. If cross-talk plays a minor role and in the absence of movement artifacts, both bipolar potential and monopolar current measurements are suited to study changes in intermuscular coherence as an indicator of varying levels of intermuscular synchronization between different conditions.

## Data Availability Statement

The data (raw data set/processed data sheet) for this study can be found in the Mendeley data repository (Mohr et al., 2018).

## Author Contributions

MM and VvT conceived and designed the experiments. TS performed the experiments. TS, MM, and VvT analyzed the data. VvT and BN contributed reagents/materials/analysis tools. BN supervision. MM wrote the first manuscript draft. MM, TS, VvT, and BN reviewed, edited, and accepted final manuscript version.

## Conflict of Interest Statement

The authors declare that the research was conducted in the absence of any commercial or financial relationships that could be construed as a potential conflict of interest.
